# Extraction of γ-chitosan from insects and fabrication of PVA/γ-chitosan/kaolin nanofiber wound dressings with hemostatic properties

**DOI:** 10.1186/s11671-024-04016-6

**Published:** 2024-05-02

**Authors:** Hakyong Lee, Jinkyeong Kim, Suwan Myung, Tae-Gon Jung, Dong-Wook Han, Bongju Kim, Jae-Chang Lee

**Affiliations:** 1 Research Center for Bio-Based Chemistry, Korea Research Institute of Chemical Technology (KRICT), Ulsan, 44429 Republic of Korea; 2 Department of Cogno-Mechatronics Engineering, Pusan National University, Busan, 46241 Republic of Korea; 3 Medical Device Development Center, Osong Medical Innovation Foundation, Chungju, 28160 Republic of Korea; 4 Dental Life Science Research Institute, Seoul National University Dental Hospital, Seoul, 03080 Republic of Korea

**Keywords:** Polyvinyl alcohol, γ-chitosan, Kaolin, Hemostasis, Electrospinning, Oral wound dressing

## Abstract

A nanofiber-based composite nonwoven fabric was fabricated for hemostatic wound dressing, integrating polyvinyl alcohol (PVA), kaolin, and γ-chitosan extracted from three type of insects. The γ-chitosan extracted from *Protaetia brevitarsis seulensis* exhibited the highest yield at 21.5%, and demonstrated the highest moisture-binding capacity at 535.6%. In the fabrication process of PVA/kaolin/γ-chitosan nonwoven fabrics, an electrospinning technique with needle-less and mobile spinneret was utilized, producing nanofibers with average diameters ranging from 172 to 277 nm. The PVA/kaolin/γ-chitosan nonwoven fabrics demonstrated enhanced biocompatibility, with cell survival rates under certain compositions reaching up to 86.9% (compared to 74.2% for PVA). Furthermore, the optimized fabric compositions reduced blood coagulation time by approximately 2.5-fold compared to PVA alone, highlighting their efficacy in hemostasis. In other words, the produced PVA/kaolin/γ-chitosan nonwoven fabrics offer potential applications as hemostatic wound dressings with excellent biocompatibility and improved hemostatic performance.

**Graphical abstract:**

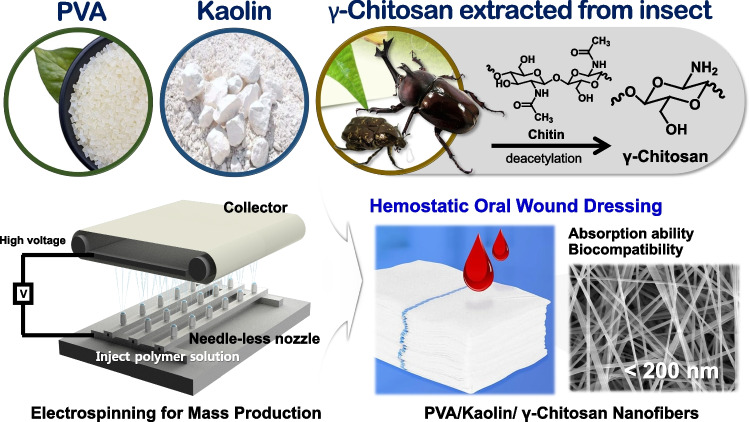

**Supplementary Information:**

The online version contains supplementary material available at 10.1186/s11671-024-04016-6.

## Introduction

Wound dressings are crucial medical materials for protecting wounds, promoting healing, preventing infections, and ensuring patient comfort [1]. These dressings are crafted from biocompatible materials like polylactide (PLA), polycaprolactone (PCL), polyvinyl alcohol (PVA), and others, to minimize adverse health impacts [2–4]. PVA, recognized for its chemical stability, high water solubility, and excellent biodegradability, is widely used in medical care system, having gained approval from the U.S. FDA for biomedical use [5–7]. It forms the basis of many wound dressing innovations, including nonwoven fabrics that offer essential oxygen permeability and moisture retention [8]. The development of PVA-based wound dressings with antimicrobial, drug-loading, and biodegradable features has achieved significant progress in the field of wound care [9, 10]. Among these advancements, the exploration of hemostatic wound dressings by loading active factors remains a major field of interest in contemporary medical research [11–13].

Kaolin is a mineral component with stable thermal properties that is non-toxic, non-allergenic and non-immunogenic. The surface of kaolin consists of layers with tiny pores that offer excellent adsorption properties. Owing to this structure, kaolin can effectively absorb moisture from blood, and it activates Hageman factor (factor XII) when it contacts blood, thus promoting efficient coagulation [14]. Factor XII is an essential component of the intrinsic coagulation pathway, and kaolin plays an indirect role in facilitating the blood clotting process where factor XII is involved. Kaolin's ability to absorb moisture and concentrate blood components makes it a valuable component in hemostatic applications, aiding in the efficient and rapid control of bleeding. For these reasons, kaolin can be used as a hemostatic and healing agent, particularly for small wounds or environments where disinfection is challenging. It can be beneficial in emergency situations where bleeding control is particularly important.

Chitosan, a derivative of chitin, is highly valued in clinical applications for its biodegradability, biocompatibility, antimicrobial, and anti-inflammatory properties [15]. Its structure, comprising linear amino polysaccharides (with repeating units of d-glucosamine and N-acetyl-d-glucosamine), makes it effective in wound dressings to prevent infections and reduce inflammation. Notably, its antimicrobial efficacy against pathogens including gastrointestinal bacteria and norovirus have been widely reported, such as its use as an antibiotic against microorganisms and its utility as a starvation-based antimicrobial agent [16–18]. Chitosan, generally classified as α-, β-, and γ- types from marine (shrimp shells and squid bones) and insect origins, shows diverse properties for various applications. The α-, and β- chitosan are valued for its high molecular weight and stability, suitable for medical, food, and cosmetic uses due to its durability [19]. On the other hand, insect-derived γ-chitosan with a lower molecular weight and higher absorption is preferred for pharmaceutical uses, particularly drug delivery, because of its rapid interaction with biological systems [20]. This variability of γ-chitosan not only addresses environmental and resource issues of marine extraction but also reduces side effects like allergies, enhancing its suitability for sensitive medical applications [21, 22]. Falabella et al. investigated the antimicrobial activity of γ-chitosan produced from insects, reporting a new perspective for use in the biological fields [23].

Recent advances have improved material functionality by converting drug factors into nanostructures or blending them with other substances, leading to the development of nanofiber structures through nanotechnology [24, 25]. Nanofiber structures offer several advantages such as increased surface area, enhanced polymer material characteristics, more effective drug delivery, enhanced wound penetration and adaptability, as well as improved fluidity and breathability [26]. One technique used to manufacture nanofibers is electrospinning, which produces nanofibers via electrostatic forces generated by high voltage. This method typically produces fibers with diameters of 100‒500 nm. Appropriate proportions of functional drug factors can be mixed and added to polymer solutions during electrospinning. This allows the development of wound dressings that enable stable drug delivery over a specified period and offers potential for wound healing applications. Research utilizing electrospinning has been reported in the field of antibacterial mats, patches for rapid hemostasis, wound dressings, drug delivery systems, as well as tissue engineering, using various biocompatible polymers such as PLA, PCL, PLGA, PEO and PVA [27–30]. Among these, PVA-based nanofibers are particularly advantageous in developing wound dressings containing hemostatic factors, releasing them effectively at the wound site and promoting healing due to their hydrophilic nature and high specific surface area of the nanofiber structure.

In this study, we manufactured hemostatic wound dressings using a nanofiber nonwoven fabric that includes kaolin and γ-chitosan as active drug components mixed with PVA. First, the γ-chitosan was extracted from three type of insects and compared its structure and properties with those of commercially available chitosan. By incorporating drug-loaded particles, kaolin and γ-chitosan with PVA, nanofiber-based PVA/kaolin/γ-chitosan nonwoven fabric mats were prepared. To enable effective mass production, the needle-less electrospinning technique was adopted, diverging from conventional methods that involve surface spraying or coating with drug-loaded particles. This study stands out for its innovative material combination and the novel use of insect-derived γ-chitosan in creating biocompatible and effective wound care solutions. While there are commonalities in terms of techniques and general objectives (such as fabricating biocompatible and hemostatic materials) [31], the unique combination of materials (PVA, γ-chitosan, and kaolin), especially the use of γ-chitosan from insects, sets this study apart.

## Methods

### Materials

The following were sourced from the respective suppliers: *Protaetia brevitarsis seulensis* (white-spotted flower chafer), *Allomyrina dichotoma* (rhinoceros beetle), and *Eophileurus chinensis* (rhinoceros beetle larvae) (TFIF Korea, Ulsan, Korea); PVA (22–82-S2) (Kuraray Co., Ltd., Tokyo, Japan). Kaolin (Al_2_Si_2_O_5_(OH)_4_), D-glucuronic acid (98%), chitosan from shrimp shells (α-chitosan, 75%), hydrochloric acid (HCl, 35‒37%), and sodium hydroxide (NaOH, 97%) (Sigma-Aldrich Corp, St. Louis, MO, USA); Soybean oil (99.9%, 876.38 g/mol, CJ Cheiljedang Co., Seoul, Korea). All reagents were used without further purification.

### Extraction process of γ-chitosan from insects

The chitin was extracted from *P. brevitarsis seulensis*, *A. dichotoma*, and *E. chinensis* then converted it into γ-chitosan via deacetylation according to procedures reported in previous literature [32, 33]. First, the frozen insects were thawed at room temperature for 2 h, washed with distilled water, dried at 50 °C for 24 h, ground into a uniform powder and passed through a 500 µm sieve. The powder was chitinized by mixing it with 1 M HCl at 100 °C in an oil bath for 30 min. The resulting powder was filtered and washed with distilled water until the pH reached 7.0. Protein was removed by reacting the chitin powder with 1 M NaOH at 80 °C for 24 h. The chitin was washed with distilled water until the pH reached 7.0, then the chitin was vacuum-dried at 50 °C for 24 h. The chitin was modified into γ-chitosan by deacetylation at 90 °C for 9 h in 55% NaOH: distilled water (1:20 w/v). The deacetylated chitin was washed with distilled water until it reached pH 7.0 then three types of γ-chitosan were generated by vacuum drying at 50 °C. The chitin and γ-chitosan yields were calculated as:

1 $$Chitin yield \left(\%\right)=(weight of chitin/weight of insect powder)\times 100$$

2 $$Chitosan yield \left(\%\right)=\left(weight of chiosan/weight of chitin\right) \times 100$$

### Preparation of PVA/kaolin/γ-chitosan solution

Mixtures of various composition ratios were prepared to manufacture nonwoven fabric based on PVA/kaolin/γ-chitosan. We prepared PVA:kaolin weight ratios of 90/10, 80/20, 70/30, and 60/40. The nomenclature for PVA:kaolin samples based on wt% of kaolin was PVA_K_n_. Table 1 shows the solution preparation conditions. For instance, PVA_K_10_ was prepared as follows. Sieved kaolin (63 μm) powder (5.6 g) was dispersed in distilled water (300 mL), then stirred at 70 °C for 12 h with PVA (40.9 g) to produce 12 wt% PVA/kaolin. A similar method was applied to prepare the PVA/kaolin/γ-chitosan, with the initial step involving the dissolution of γ-chitosan in a 2% D-gluconic acid. Specifically, 0.6 g of γ-chitosan extracted from insects was added to 2% D-gluconic acid (300 mL) to create a 0.2 wt% γ-chitosan solution. Kaolin and PVA were subsequently added to prepare a PVA/kaolin/γ-chitosan solution, and samples containing γ-chitosan were named PVA_K_n_Cs. All solutions prepared before electrospinning were ultrasonicated at 40 °C for 1 h to enhance particle dispersion.

**Table 1:** Preparation of PVA/kaolin/γ-chitosan nonwoven nanofabrics

Sample	Content of raw materials			Electrospinning conditions	
	PVA (wt%)	Kaolin (wt%)	γ-Chitosan (phr)	Voltage (kV)	TCD (cm)
PVA	100	–	–	38	13
PVA_K_10_	90	10	–	34	12
PVA_K_20_	80	20	–	34.3	12
PVA_K_30_	70	30	–	33.8	12
PVA_K_40_	60	40		33.3	10.5
PVA_Cs	100	–	0.2	39.2	12
PVA_K_10_Cs	90	10	0.2	39.7	11
PVA_K_20_Cs	80	20	0.2	38.4	11
PVA_K_30_Cs	70	30	0.2	39.3	11
PVA_K_40_Cs	60	40	0.2	39.5	11

### Preparation of PVA/kaolin/γ-chitosan nonwoven nanofabrics using electrospinning

**Fig. 1:**
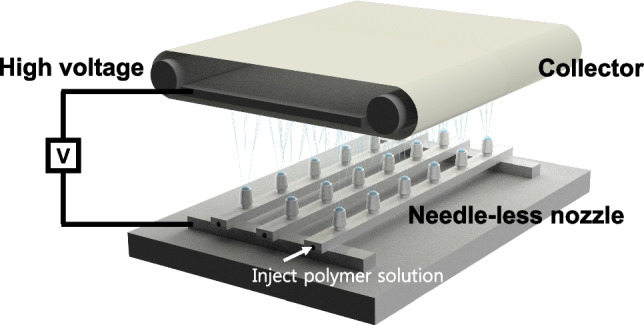
Electrospinning device equipped with needle-less and mobile spinneret

Figure 1 shows a diagram of the electrospinning setup. The electrospinning device is configured with an upward-facing spinning nozzle, allowing the solution to pool inside the wineglass-shaped nozzle, thereby enabling the solution to maintain a stable spherical form. The electrospinning device allowed the solution inside the nozzle to exist in a spherical form. Applying voltage enabled the solution, mixed with functional organic and inorganic particles such as kaolin and γ-chitosan, to be spun smoothly without clogging the nozzle. The setup includes multiple nozzles (0.5 mm diameter, 0.18 mL dosing volume for each) mounted on reciprocating movable spinning electrode (45 cm in length), with adjustable spacing between them to avoid the influence of electrostatic forces. To produce a uniform nonwoven nanofabrics, the distance between each nozzle was set to 8 cm, mounting a total of 24 nozzles across 6 spinning electrodes. The dosing rate for each nozzle was set to 25 µL/min, and the spinning was conducted for 1 h. The nanofibers were collected on a polypropylene (PP) base nonwoven fabric (50 × 200 cm) mounted on a cyclical substrate roller, with the rewinding speed set to 3 m/min. The high viscosity of the electrospinning solution due to the addition of kaolin and γ-chitosan reduces spinning stability, so to compensate for this, voltage and tip-to-collector distance (TCD) were adjusted to optimize the spinning conditions for each sample. Table 2 summarizes the optimal electrospinning conditions for each sample, including voltage and TCD, necessary to manufacture the nonwoven fabric composed of PVA/kaolin/γ-chitosan nanofibers. The fabric was dried in a vacuum oven at 50 °C for 24 h to completely evaporate the residual solvent, sterilized under ultraviolet irradiation for 6 h, and the PP base nonwoven fabric was removed for analysis and evaluation.

**Table 2:** Characteristic of chitin and γ-chitosan depending on insect type

Source	Yield (%)			γ-Chitosan	
	Chitin extracted from insects	γ-Chitosan derived from chitin	γ-Chitosan derived from insects	Water binding capacity (%)	Fat-binding capacity (%)
*P. brevitarsis seulensis*	29.2	74.8	21.5	535.6	152.1
*A. dichotoma*	15.3	72.3	10.8	497.9	151.6
*E. chinensis*	10.4	69.1	6.9	415.9	229.7

### Measurements

#### Water/fat-binding capability

A conical tube containing chitosan was placed on a balance, and water/soybean oil was added. The mixture was vigorously stirred for 1 min. The mixture was then stabilized at room temperature (25 °C) for 12 h and centrifuged at 6000 rpm; then, the supernatant was discarded, and the pelleted material was weighed. This process was repeated three times, and the average value was calculated. The moisture and lipid binding capacity were determined as [34]:

3 $$Water-binding capability \left(\%\right)= \frac{Water bound (g)}{Sample bound (g)} \times 100$$

4 $$Fat-binding capability \left(\%\right)= \frac{Oil bound (g)}{Sample bound (g)} \times 100$$

#### Two-dimensional wide-angle X-ray diffraction findings

Chitosan structure was determined by two-dimensional wide-angle X-ray diffraction (2D-WAXD) using a D/MAX RAPID II X-ray diffractometer (Rigaku, Corp., Tokyo, Japan) under 40 kV and 30 mA for 15 min with a rotation speed of 0.3 mm and 2°/s.

#### Fourier-transform infrared spectroscopy

We confirmed structural changes in chitosan resulting from the deacetylation of chitin extracted from insects using Fourier transform infrared spectroscopy (FT-IR) in attenuated total reflectance mode. Changes were measured using the Nicolet 6700 FT-IR spectrometer (Thermo Fisher Scientific Inc., Waltham, MA, USA) at a wavelength range of 600‒4000 cm^−1^ with 16 scans and a resolution of 4 cm^−1^.

#### Field-emission scanning electron microscopy

We compared the surface morphology of the nanofibers by coating them with platinum using a Q150T sputter coater (Quorum Technologies, Laughton, UK) for 90 s at 15 mA. The nanofiber surface was visualized using a field-emission scanning electron microscope (FE-SEM), MIRA3 LMH Inbeam detector (TESCAN, Brno-Kohoutovice, Czech Republic). Changes in nanofiber diameter measured using ImageJ software (US National Institutes of Health, Bethesda, MD, USA), are presented as distribution determined by measuring the average diameter of 100 nanofibers. The pore size and porosity of the nanofibers were also measured using ImageJ.

#### Scanning electron microscopy with energy-dispersive X-ray spectroscopy

We compared the overall dispersion state of the nanofiber surface and the kaolin particle content in conjunction with X-ray spectroscopy. The surfaces of samples were sputter-coated with platinum, analyzed by energy-dispersive X-ray spectroscopy (SEM–EDS, MIRA 3 LMH Inbeam detector), and mapped.

#### Thermogravimetric analysis

The inorganic content of PVA/kaolin and PVA/γ-chitosan/kaolin nanofibers was measured using a thermogravimetric analyzer (TGA), Q500 (TA Instruments, New Castle, DE, USA) with heating increments of 10 °C/min from 25 to 600 °C under a nitrogen atmosphere.

#### Cell culture

L929 murine fibroblast cells (American Type Culture Collection (Manassas, VA, USA) were routinely cultured in high-glucose Dulbecco’s modified Eagle medium (DMEM) (Welgene, Daegu, Korea) containing 10% fetal bovine serum (Welgene) and 1% antibiotic–antimycotic (10,000 U penicillin, 25 µg/mL amphotericin B, and 10 mg streptomycin (Sigma-Aldrich Corp.) in humidified incubator at 37 °C under a 5% CO_2_ atmosphere. The cells were subcultured at 70% confluence using a trypsin–EDTA (Sigma-Aldrich). Cells from passages 3 and 4 were seeded at an optimized density in each culture plate.

#### Cytocompatibility assessment

Ten nonwoven nanofabric membranes (1 × 6 cm) were shaken at 80 rpm in 10 mL of culture medium at 37 °C for 72 h in a humidified incubator. The extract was sterilized under ultraviolet radiation overnight, then added to 96-well plates (n = 4) containing 5 × 104 L929 cells/mL. Cells were reacted with extracts for 24 h and the cell viability was determined using Cell Counting Kit-8 (CCK-8; Dojindo, Kumamoto, Japan). The culture medium was replaced with 180 µL DMEM and 20 µL of CCK-8 reagent. Supernatants (100 µL) were incubated at 37 °C in 96-well plates for 2 h, then absorbance at 450 nm was determined using a Varioskan LUX microplate reader (Thermo Fisher Scientific Inc.). The cells were observed under an optical microscope (Leica DMIL, Leica Microsystems, Wetzlar, Germany). For live and dead assay, 2 µM calcein AM and 4 µM ethidium homodimer-1 (Invitrogen, Waltham, MA) were treated for 30 min in an incubator at 37 °C in the dark, and the images were obtained by fluorescence microscopy (IX81, Olympus, Tokyo, Japan).

#### Blood clotting time

The Thrombin test was conducted to observe the blood coagulation ability of nonwoven nanofabrics. Nonwoven nanofabrics were cut into 1 × 1 cm pieces, and two pieces per group were attached to the slide with double-sided tape. Blood was collected from the hearts of Sprague–Dawley rats (male, 7 wk-old, 220–270 g, orientbio, gapyeong, Korea). The time from when 0.05 ml of blood was dropped on each nonwoven nanofabrics sample until the blood coagulated was measured to compare and observe the time each nonwoven nanofabrics sample had on blood coagulation. Pharyngeal pumping rate of nematodes treated with different fabrics eluates was carried out as described in [35]. This test was made in triplicate and about 40 worms were considered in each experiment.

#### Statistical analysis

All experiments were performed in triplicate. All data were processed using the SigmaPlot 14 statistical program (Systat Software Inc., San Jose, CA, USA) and expressed as mean ± standard deviation (SD). In addition, the data were evaluated using the Mann–Whitney rank-sum test. The results were considered significant if the *p* value was less than 0.05.

## Results and discussion

### Extraction and characteristics of γ-chitosan

Insects typically consist of 25‒40% fat, 30‒40% protein, and 10‒20% chitin, that is abundant in their exoskeletons and can be converted into γ-chitosan [36]. Therefore, we extracted γ-chitosan from powdered insects by chemical demineralization, deproteinization, and deacetylation. Table 2 shows that the overall yields of γ-chitosan derived from insects were 21.5% 10.8% and 6.9% from *P. brevitarsis seulensis*, *A. dichotoma*, and *E. chinensis*, respectively. Most of the chitin in the *P. brevitarsis seulensis* exoskeleton with the highest mass ratio was converted into chitosan. This indicates that *P. brevitarsis seulensis* is potentially the most efficient species for γ-chitosan extraction, particularly due to its high chitin content and conversion ratio.

**Fig. 2:**
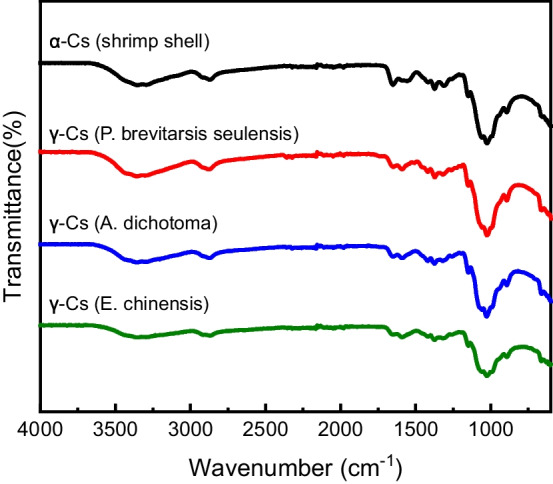
Fourier transformed infrared spectra of α-chitosan and γ-chitosan

We confirmed and compared structural changes between chitin and γ-chitosan by analyzing FT-IR spectra. Figure 2 shows the FT-IR spectra of γ-chitosan extracted from each insect and commercial α-chitosan. The major peaks of interest in the chitosan FT-IR spectrum corresponded to the amino and carbonyl groups. Deacetylation decreased the peak intensity of the carbonyl group (C=O stretching, 1647‒1654 cm^−1^) in chitin, and a new peak corresponding to the amino group (-NH stretching, 1589 cm^−1^) emerged. Peaks related to the hydroxyl (–OH stretching) and methylene (–CH_2_^−^ stretching) groups within chitosan were found at 3350 cm^−1^ and 1420 cm^−1^, respectively. The γ-chitosan extracted from insects and commercial α-chitosan did not significantly differ.

**Fig. 3:**
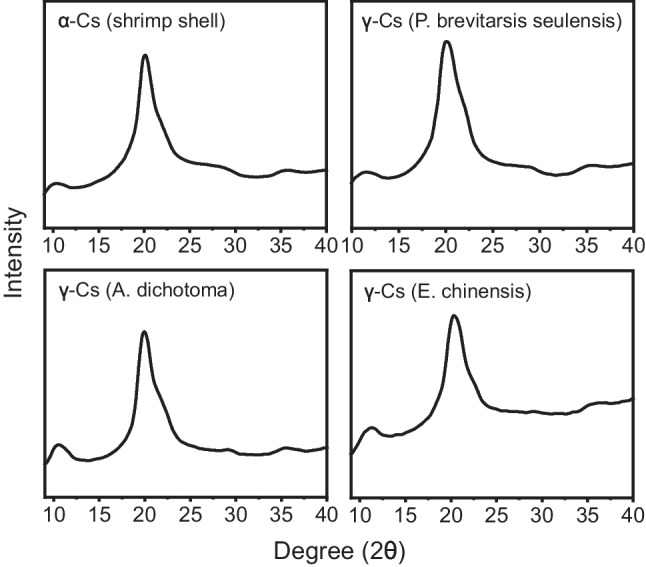
Two-dimensional wide-angle X-ray diffraction spectra of α-chitosan and γ-chitosan

The crystal structures of α-chitosan from shrimp shells and γ-chitosan from the three types of insects were compared using 2D-WAXD to assess the potential substitution of insect-derived γ-chitosan for commercial α-chitosan (Fig. 3). Representative peaks for chitosan were found at 10° and 20° for all samples [37]. The crystal structures of α-chitosan and the three γ-chitosan samples were remarkably similar. These findings further emphasize the suitability of *P. brevitarsis seulensis* as a viable alternative for commercial α-chitosan production. These results indicated that insect-derived γ-chitosan has the potential to replace commercial chitosan.

The moisture and fat-binding abilities of the insect-derived γ-chitosan were measured, and Table 2 present their results. The moisture binding abilities of γ-chitosan from *P. brevitarsis seulensis*, *A. dichotoma*, and *E. chinensis* were 535.6%, 497.8%, and 415.8%, respectively, and their fat-binding abilities were 152.0%, 151.5%, and 229.6%, respectively. Materials that can efficiently absorb and retain moisture can maintain wounds in an appropriate state, while reducing inflammation and promoting healing. Among the three types of insects, *P. brevitarsis seulensis* had the most moisture binding ability and the highest yield. Therefore, γ-chitosan extracted from *P. brevitarsis seulensis* was used to manufacture a PVA/kaolin/γ-chitosan nanofiber nonwoven fabric.

### Manufacturing and morphology of PVA/kaolin/γ-chitosan nanofiber nonwoven fabrics

**Fig. 4:**
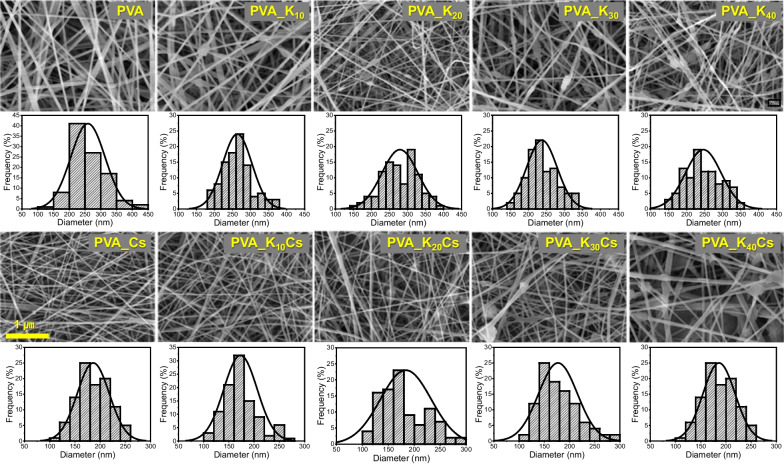
SEM images of PVA/kaolin/γ-chitosan nonwoven nanofabrics

Various combinations of PVA/kaolin/γ-chitosan nanofiber nonwoven fabrics were manufactured using an electric spinning device equipped with a wineglass shaped nozzle. In the electrospinning process, numerous parameters influence the final morphology of nanofibers, including material properties (molecular weight, concentration, viscosity, dielectric constant, surface tension), spinning conditions (voltage, flow rate, spinning distance), and environmental conditions (temperature, humidity) [38, 39]. Figure 4 and show the morphology of the PVA/kaolin/γ-chitosan nanofiber nonwoven fabrics manufactured under optimal spinning conditions for each sample determined by FE-SEM. The PVA spinnability resulted in the formation of uniform fiber structures in all samples. The average diameters of the nanofibers (nm) were 259 ± 55 (PVA), 264 ± 43 (PVA_K_10_), 277 ± 47 (PVA_K_20_), 237 ± 42 (PVA_K_30_), 246 ± 50 (PVA_K_40_), 187 ± 34 (PVA_Cs), 172 ± 30 (PVA_K_10_Cs), 185 ± 50 (PVA_K_20_Cs), 185 ± 55 (PVA_K_30_Cs), and 187 ± 34 (PVA_K_40_Cs). Satapathy et al. and Li et al. reported on the characteristics of a nanofiber-based drug delivery scaffold with diameters ranging from 150 to 380 nm by optimally controlling the morphology, and we obtained nanofiber nonwoven fabrics with a similar structure [5, 33]. The tendency of the fiber diameter to decrease upon the addition of γ-chitosan was attributed to the influence of the amino (–NH_2_) and ketone (–CO–) groups in the chitosan structure affecting the electrical conductivity and electrostatic properties of the mixture [40]. Despite concentrations up to 40 wt%, kaolin particles were well dispersed within the nanofiber nonwoven fabric without aggregation. The pore diameters of all samples were less than 1 µm, and the porosity was relatively consistent, ranging from 36.2 to 39.8% (Fig. S1).

The SEM–EDS results in Table 3 clearly show differences in particle composition and dispersion on the nanofiber surfaces. The elemental composition of aluminum and silicon increased along with the kaolin content, indicating the uniform dispersion of kaolin particles within the nanofibers even at high concentrations. Figure S2 present carbon, oxygen, aluminium, and silicon distribution image on the nanofiber surfaces through EDS mapping. Unlike traditional electrospinning devices with needle-type nozzles, the nozzle of our device did not become clogged, and mass production of nanofiber nonwoven fabrics was achieved using several nozzles at the same time. Namely, the experimental results showed that the electrospinning nanofibers can be stably manufactured in mass scale using a needle-less and mobile spinneret.

**Table 3:** Surface components of PVA/kaolin/γ-chitosan nonwoven nanofabrics

Element	PVA		PVA_K_10_		PVA_K_20_		PVA_K_30_		PVA_K_40_	
	Element (wt%)	Atom (%)	Element (wt%)	Atom (%)	Element (wt%)	Atom (%)	Element (wt%)	Atom (%)	Element (wt%)	Atom (%)
C K	45.3	52.5	43.3	52.5	41.1	51.2	27.8	37.0	21.0	29.5
O K	54.7	47.5	46.4	42.1	42.6	39.9	50.6	50.5	50.2	52.9
Al K	-	-	4.0	2.2	7.1	4.0	9.1	5.4	12.5	7.8
Si K	-	-	6.3	3.2	9.2	4.9	12.5	7.1	16.3	9.8
Total	100.0	100.0	100.0	100.0	100.0	100.0	100.0	100.0	100.0	100.0

Element	PCs		PVA_K_10_Cs		PVA_K_20_Cs		PVA_K_30_Cs		PVA_K_40_Cs	
	Element (wt%)	Atom (%)	Element (wt%)	Atom (%)	Element (wt%)	Atom (%)	Element (wt%)	Atom (%)	Element (wt%)	Atom (%)
C K	24.6	30.3	42.6	50.5	41.3	49.7	37.1	46.8	19.5	28.6
O K	75.4	69.7	53.1	47.3	51.8	46.7	47.0	44.5	43.5	47.8
Al K	-	-	1.5	0.8	2.9	1.5	6.7	3.7	15.2	9.9
Si K	-	-	2.8	1.4	4.0	2.1	9.2	5.0	21.8	13.7
Total	100.0	100.0	100.0	100.0	100.0	100.0	100.0	100.0	100.0	100.0

*C* carbon; *O* oxygen; *Al* aluminum; *Si* silicon; *K* potassium

### Thermal analysis of PVA/kaolin/γ-chitosan nanofiber nonwoven fabrics

**Fig. 5:**
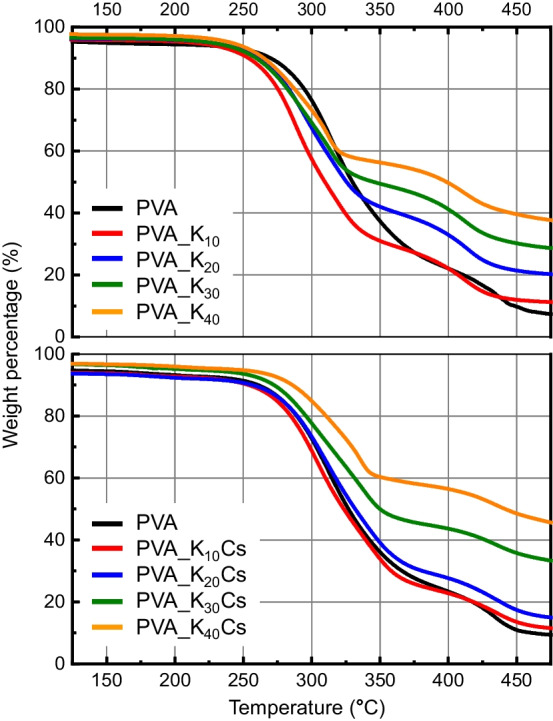
Thermogravimetric curves of PVA/kaolin/γ-chitosan nonwoven nanofabrics

We analyzed residual inorganic contents after the thermal decomposition of PVA/kaolin/γ-chitosan nanofiber nonwoven fabrics. Figure 5 shows the decomposition behavior and residual content of each sample. The alterations in the TGA curve slopes are attributed to the multi-stage decomposition process, which includes the dehydration, depolymerization, and decomposition of organic and inorganic components. Since kaolin and chitosan completely decompose at 1200 °C, measuring the residual content at a specific temperature (600 °C) allows the determination of the inorganic contents of PVA/kaolin/γ-chitosan [41]. As depicted in Fig. 5, all PVA, PVA_K_n_, and PVA_K_n_Cs samples exhibited pronounced two-stage decomposition behaviors. The first weight loss occurred between 250 and 360 °C, related to the removal of hydroxyl groups from PVA and the formation of polyene macromolecules [41]. The weight loss for the PVA was more significant than for PVA_K_n_ and PVA_K_n_Cs, and a clear reduction in weight loss degree was observed with an increase in kaolin concentration. Specifically, the thermal decomposition residue content for each sample was as follows: 6.08% (PVA), 10.98% (PVA_K_10_), 19.57% (PVA_K_20_), 27.60% (PVA_K_30_), 36.15% (PVA_K_40_), 10.47% (PVA_K_10_Cs), 13.49% (PVA_K_20_Cs), 30.37% (PVA_K_30_Cs), and 41.76% (PVA_K_40_Cs). Consequently, the increasing residual inorganic content with higher kaolin content indicated a uniform kaolin dispersion within the nanofiber nonwoven fabric.

### Cellular toxicity assessment

**Fig. 6:**
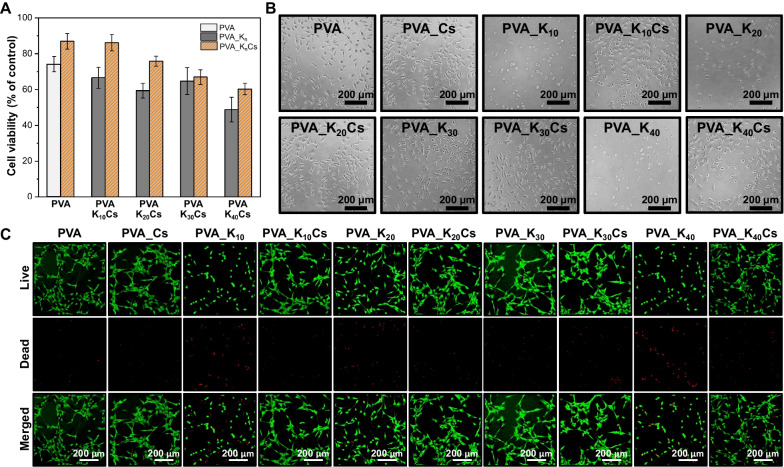
Cell viability of PVA/kaolin/γ-chitosan nonwoven nanofabrics after 24 h of incubation

Cellular toxicity tests play a crucial role in evaluating the safety, biocompatibility, and potential negative impact of oral wound dressings on human cells. Therefore, we measured cell viability and proliferation rates in response to changes in extract concentrations using CCK-8 analysis (Fig. 6B), optical microscopy (Fig. 6B), and live and dead cell assay (Fig. 6C) to compare the influence of PVA/kaolin/γ-chitosan nanofiber nonwoven fabrics on cell proliferation. Figure 6 shows the cell proliferation rates in the presence of PVA, PVA/kaolin, and PVA/kaolin/γ-chitosan nanofiber nonwoven fabrics relative to an extract concentration of 100%. The cell survival rate under PVA alone was 74.2%, and slightly decreased as the kaolin concentration increased. The excessive dose of kaolin can lead to its internalization into cells, resulting in the production of reactive oxygen species and subsequent DNA damage [42, 43].

Adding γ-chitosan increased the cell survival rate, possibly because of its antioxidant/antibacterial effects. Cell survival rates were respectively 86.1% and 86.9% under PVA_K_10_Cs and PVA_K_20_Cs, indicating excellent biocompatibility. The dose-dependent cytotoxicity induced by kaolin and the counteractive influence of γ-chitosan can be additionally verified through microscopic imaging and live and dead cell assays (Fig. 6B and C). The images suggest that the inclusion of γ-chitosan preserved the natural fibroblastic morphology of the L929 cells, particularly evident in the higher concentration groups. Thus, the introduction of appropriate amounts of kaolin and chitosan is essential to the development of biocompatible oral wound dressings. The high cell survival rates indicated that these materials exert minimal negative effects on cells, thus rendering them suitable as biocompatible dressings for oral wounds.

### Hemostatic assessment

**Fig. 7:**
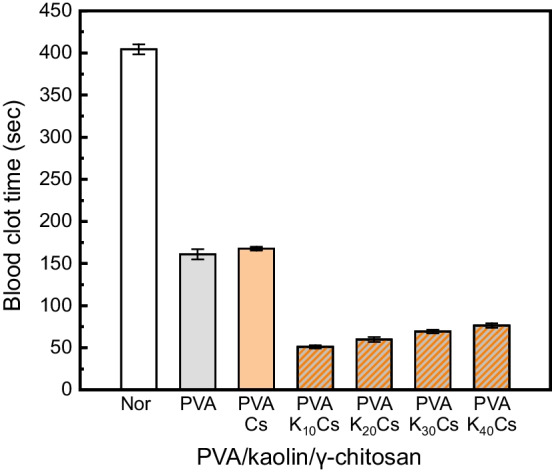
Blood clotting time of PVA/kaolin/γ-chitosan nonwoven nanofabrics

Hemostatic wound dressings serve a vital role in controlling bleeding at wound sites, aiding blood clotting, and preventing infections from various harmful agents. Specifically, in PVA/kaolin wound dressings, kaolin is acknowledged as a powerful localized hemostatic agent that effectively stimulates the clotting process. Kaolin, mainly composed of the mineral kaolinite and aluminum silicate, impacts blood clotting significantly due to its surface's negative charge. When in direct contact with blood, kaolin promotes the activation of factor XII and platelets, initiating the coagulation pathway [41]. Therefore, various PVA/kaolin/γ-chitosan nanofiber nonwoven fabrics were manufactured with different amounts of kaolin, and hemostasis was assessed in those with the most favorable biocompatibility. Figure 7 shows blood clotting times. The initial blood clotting times for PVA and PVA_Cs nanofiber nonwoven fabrics were similar at 161 and 167 s. This suggested that γ-chitosan does not influence blood clotting. In contrast, blood clotting times ranged from 51 to 76 s for PVA_K_n_Cs, indicating an improved hemostatic effect. This was attributed to even kaolin dispersion in PVA, which is a highly effective hemostatic agent. The kaolin content increased and the blood clotting time notably increased slightly. This phenomenon was due to the relatively hydrophobic nature of kaolin, which is an aluminum silicate mineral that does not readily dissolve in water. As the kaolin content increased, its affinity for blood decreased [28]. Therefore, we determined that the optimal kaolin content for hemostasis was 10‒20 wt%, resulting in a blood clotting time that was ~ 2.5-fold shorter than that of PVA alone. These results are consistent with the findings of Meng et al., who investigated wound dressings based on PEO incorporating kaolin and α-chitosan, showing similar hemostatic effects [44]. In conclusion, the PVA/kaolin/γ-chitosan nanofiber nonwoven fabric, with a controlled kaolin content, had an enhanced hemostatic effect, indicating its potential as an improved hemostatic wound dressing.

## Conclusion

This research presents an applicable approach in the field of oral wound dressing through the fabrication of PVA/kaolin/γ-chitosan nanofiber nonwoven fabrics, leveraging a unique combination of materials and manufacturing techniques. We have innovatively integrated γ-chitosan derived from *P. brevitarsis seulensis*, which has shown the highest yield and moisture absorption rate, with kaolin in a PVA matrix. This integration was achieved using a needle-less, mobile spinneret for electrospinning, allowing for controlled nanofiber diameters and effective dispersion of functional particles. Our comprehensive analysis revealed that these nanofibers exhibit enhanced biocompatibility, and potential hemostatic properties compared to control groups. Specifically, the antimicrobial properties of γ-chitosan, coupled with the hemostatic influence of kaolin, significantly improved cell survival rates and reduced blood coagulation times by 2.5-fold. Although it is need to further studies for determination of the hemostatic effect including in vivo test, this approach, while strongly pretesting in nature for developing oral dressing products, introduces novel material properties and fabrication techniques, anticipating a new standard for future wound care in dentistry. Particularly, the structural and compositional similarities between insect-derived γ-chitosan and commercial α-chitosan highlight the potential to replace traditional marine-sourced chitosan, offering a sustainable and efficient alternative for medical material applications.

### Supplementary Information

Supplementary Information (DOCX 1870 KB)

## Data Availability

All data generated or analysed during this study are included in this published article.
