# Supplementary material for: Extraction of γ-chitosan from insects and fabrication of PVA/γ-chitosan/kaolin nanofiber wound dressings with hemostatic properties
Source: Discov Nano. 2024 May 2;19(1):77. doi: 10.1186/s11671-024-04016-6 (PMC11063014; doi:10.1186/s11671-024-04016-6)
Supplement: Supplementary file 1 — Supplementary Information (DOCX 1870 KB) [file 11671_2024_4016_MOESM1_ESM.docx]

**Supplementary Materials for Manuscript:**

**Manuscript:** Extraction of γ-Chitosan from Insects and Fabrication of PVA/γ-Chitosan/Kaolin Nanofiber Wound Dressings with Hemostatic Properties

**Fig S1** Pore morphology of nanofibers using ImageJ software.

**Fig S2** S2 Silicon distribution image through EDS mapping.

**
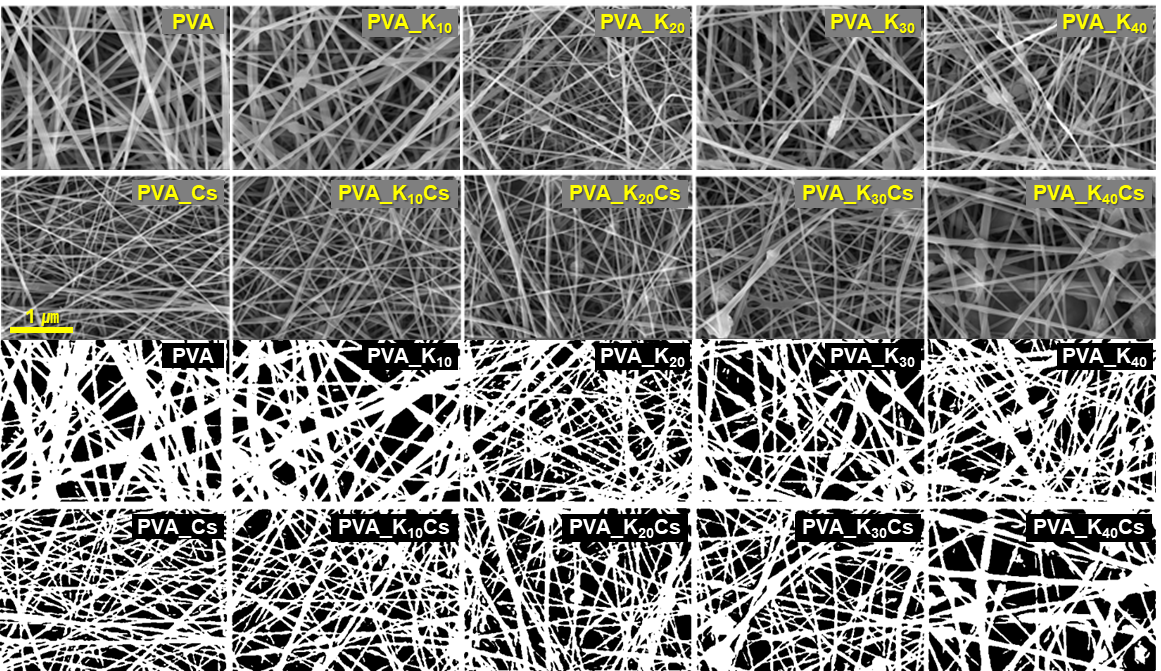
**

**Fig. S1** Pore morphology of nanofibers using ImageJ software.

**
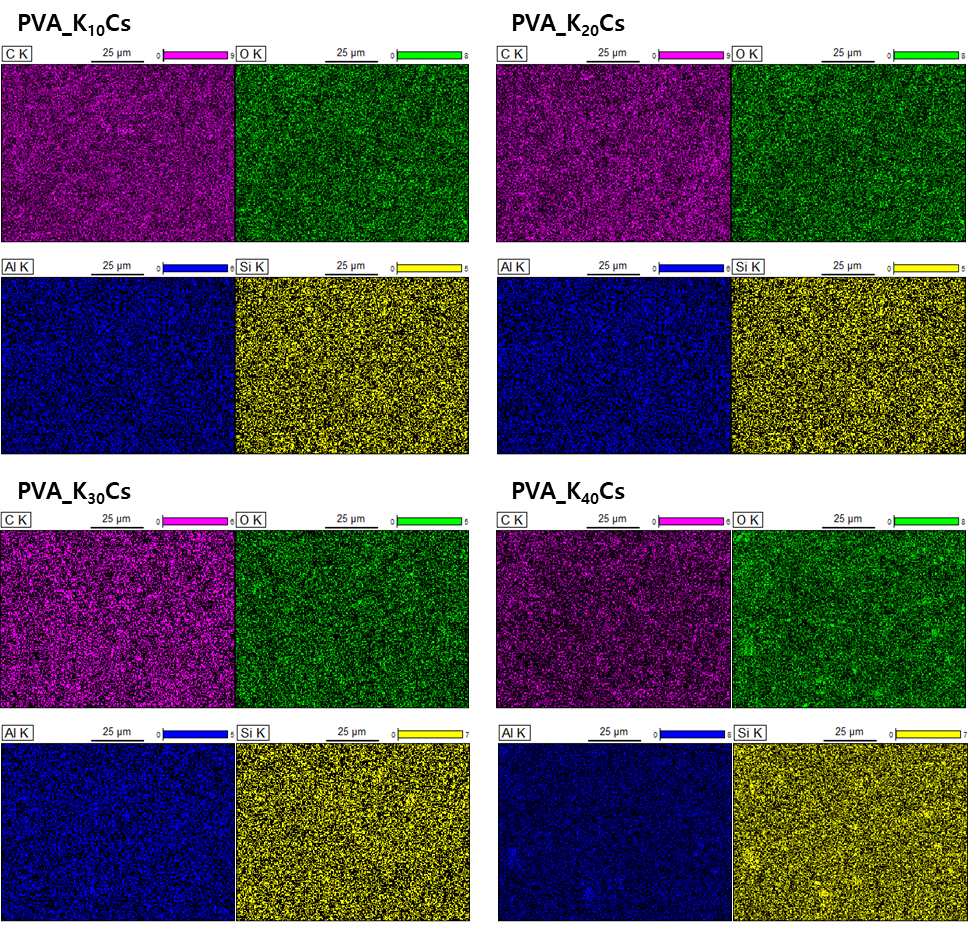
**Fig. S2 Silicon distribution image through EDS mapping.
